# Supplementary material for: HIVprotI: an integrated web based platform for prediction and design of HIV proteins inhibitors
Source: J Cheminform. 2018 Mar 9;10:12. doi: 10.1186/s13321-018-0266-y (PMC5845081; doi:10.1186/s13321-018-0266-y)
Supplement: Supplementary file 2 — Additional file 2. Source code of HIVProtI web server. [file 13321_2018_266_MOESM2_ESM.zip › HIVprotI_Source-code/hivprotI/weka-3-6-6/doc/weka/core/converters/DatabaseConnection.html]

DatabaseConnection


---


|  |  |  |
| --- | --- | --- |
| **Overview** \| **Package** \| **Class** \| **Tree** \| **Deprecated** \| **Index** \| **Help** \| **Weka's home** | |  |
| **PREV CLASS**   **NEXT CLASS** | **FRAMES**    **NO FRAMES**     **All Classes** |  |
| SUMMARY: NESTED \| FIELD \| CONSTR \| METHOD | DETAIL: FIELD \| CONSTR \| METHOD |  |


---


## weka.core.converters Class DatabaseConnection

```
java.lang.Object
  weka.experiment.DatabaseUtils
      weka.core.converters.DatabaseConnection
```

**All Implemented Interfaces:**: java.io.Serializable, RevisionHandler

---

``` public class DatabaseConnection extends DatabaseUtils ```

Connects to a database.

**Version:**
:   $Revision: 1.8 $

**Author:**
:   Len Trigg (trigg@cs.waikato.ac.nz), Stefan Mutter (mutter@cs.waikato.ac.nz)

**See Also:**: Serialized Form

---

**Field Summary**

**Fields inherited from class weka.experiment.DatabaseUtils**

`BOOL, BYTE, DATE, DOUBLE, EXP_INDEX_TABLE, EXP_RESULT_COL, EXP_RESULT_PREFIX, EXP_SETUP_COL, EXP_TYPE_COL, FLOAT, INTEGER, LONG, PROPERTY_FILE, SHORT, STRING, TEXT, TIME`


**Constructor Summary**

`DatabaseConnection()`
  
          Sets up the database drivers


| **Method Summary** | |
| --- | --- |
| `java.sql.DatabaseMetaData` | `getMetaData()`             Gets meta data for the database connection object. |
| `java.lang.String` | `getRevision()`             Returns the revision string. |
| `int` | `getUpdateCount()`             Dewtermines if the current query retrieves a result set or updates a table |
| `boolean` | `getUpperCase()`             Check if the property checkUpperCaseNames in the DatabaseUtils file is set to true or false. |

**Methods inherited from class weka.experiment.DatabaseUtils**

`arrayToString, close, close, connectToDatabase, createExperimentIndex, createExperimentIndexEntry, createResultsTable, databaseURLTipText, debugTipText, disconnectFromDatabase, execute, experimentIndexExists, getDatabaseURL, getDebug, getKeywords, getKeywordsMaskChar, getPassword, getResultFromTable, getResultSet, getResultsTableName, getSupportedCursorScrollType, getUsername, isConnected, isCursorScrollable, isCursorScrollSensitive, isKeyword, maskKeyword, passwordTipText, processKeyString, putResultInTable, select, setDatabaseURL, setDebug, setKeywords, setKeywordsMaskChar, setPassword, setUsername, tableExists, translateDBColumnType, typeName, update, usernameTipText`

**Methods inherited from class java.lang.Object**

`equals, getClass, hashCode, notify, notifyAll, toString, wait, wait, wait`

**Constructor Detail**

### DatabaseConnection

```
public DatabaseConnection()
                   throws java.lang.Exception
```

:   Sets up the database drivers

    **Throws:**: `java.lang.Exception` - if an error occurs


**Method Detail**

### getUpperCase

```
public boolean getUpperCase()
```

:   Check if the property checkUpperCaseNames in the DatabaseUtils file is
    set to true or false.

    :   **Returns:**: true if the property checkUpperCaseNames in the DatabaseUtils file is set to true, false otherwise.

---


### getMetaData

```
public java.sql.DatabaseMetaData getMetaData()
                                      throws java.lang.Exception
```

:   Gets meta data for the database connection object.

    :   **Returns:**: the meta data. **Throws:**: `java.lang.Exception` - if an error occurs

---


### getUpdateCount

```
public int getUpdateCount()
                   throws java.sql.SQLException
```

:   Dewtermines if the current query retrieves a result set or updates a table

    :   **Returns:**: the update count (-1 if the query retrieves a result set). **Throws:**: `java.sql.SQLException` - if an error occurs

---


### getRevision

```
public java.lang.String getRevision()
```

:   Returns the revision string.

    :   **Specified by:**: `getRevision` in interface `RevisionHandler` **Overrides:**: `getRevision` in class `DatabaseUtils`
    :   **Returns:**: the revision


---


|  |  |  |
| --- | --- | --- |
| **Overview** \| **Package** \| **Class** \| **Tree** \| **Deprecated** \| **Index** \| **Help** \| **Weka's home** | |  |
| **PREV CLASS**   **NEXT CLASS** | **FRAMES**    **NO FRAMES**     **All Classes** |  |
| SUMMARY: NESTED \| FIELD \| CONSTR \| METHOD | DETAIL: FIELD \| CONSTR \| METHOD |  |


---
